# Comparative Outcomes of Frontal, Bifrontal, and Pterional Craniotomies for Resection of Large Anterior Skull Base Meningiomas

**DOI:** 10.1227/ons.0000000000001830

**Published:** 2025-11-28

**Authors:** Yash Akkara, Gwendoline Fuller, Sofia Committeri, Ananjan Ganguli, Samir Bougrine, Joe M. Das, Ramesh Nair

**Affiliations:** 1 Imperial College London School of Medicine, London, UK;; 2 Department of Neurosurgery, Imperial College London NHS Trust, London, UK

**Keywords:** Bifrontal, Pterional, Frontal, Meningioma, Skull base, Neurosurgery

## Abstract

**BACKGROUND AND OBJECTIVES:**

Large anterior skull base meningiomas are common and challenging brain tumors to resect. This study evaluates the role of the frontal, bifrontal, and pterional craniotomies on outcomes after resection.

**METHODS:**

This is a retrospective study of patients with large anterior skull base operated on at our institution between 2010 and 2024 using the frontal, bifrontal, and pterional approaches. All patients were 18 years or older and had ≥1 year of clinical follow-up data. Propensity-score matching (PSM) with a one-to-many nearest neighbor matching algorithm was used to achieve 3 groups of similar patients, based on size, grade, and preoperative Karnofsky Performance Status. The Shapiro-Wilk test, paired *T*-test, analysis of variance, and Tukey test were used to establish statistical significance. Pearson R was used to evaluate PSM success, with the log-rank test used within Kaplan-Meier analysis and multiple-linear regressions performed to evaluate predictors of outcomes.

**RESULTS:**

In total, 337 patients (140M, 197F) were included, with 80, 189, and 68 patients undergoing bifrontal, frontal, and pterional craniotomies, respectively. Patients in the bifrontal group presented with significantly higher tumor size (5.46 cm, *P* = .0021) and lower mean preoperative Karnofsky Performance Status (71.15, *P* = .0059), while patients in the frontal group reported a significantly higher tumor grade vs other groups (1.65, *P* = .0025). After PSM, the bifrontal group reported a significantly higher frequency of medical (25.0%, *P* < .0001) and surgical (22.5%, *P* < .0001) complications, alongside significantly worse cosmetic outcomes (6.25%, *P* = .0054) vs other groups. Kaplan-Meier analysis showed a significantly higher rate of minor complications in the bifrontal group (*P* = .0497), alongside reduced progression-free survival in the frontal group (*P* = .0048). Regression analyses revealed olfactory groove meningiomas and subtotal resections predicted worse outcomes for the pterional and frontal approaches, respectively.

**CONCLUSION:**

The findings suggest significant difference in baseline characteristics and operative outcomes across the 3 groups, highlighting predictive factors and risk profiles that neurosurgeons can include in perioperative planning.

**ABBREVIATIONS:**
KM: Kaplan-Meier
KPS: Karnofsky Performance Status
OS: overall survival
PSM: propensity-score matching
SMDs: standardized mean differences.

Meningiomas constitute approximately 25% of all primary intracranial neoplasms.^1^ They often develop in the anterior skull base, such as within the olfactory groove and sphenoid wing. Specifically, large anterior skull tumors refer to those measuring ≥3 cm in size^2^ and are particularly challenging to treat, primarily through open craniotomies and microsurgical resection.^3^

Unilateral frontal (referred to as “frontal”) and bifrontal craniotomies are approaches used to access anterior skull base tumors. The frontal approach entails a semicoronal incision, whereas the bifrontal approach entails a bicoronal incision to reflect the scalp. While variable, the bifrontal approach is generally used for larger midline tumors, whereas the frontal approach is often used for smaller, localized tumours.^4^ The pterional/frontotemporal craniotomy involves a semicoronal arcuate incision posterior to the pterion, often used for tumors located more laterally.^5^ Nevertheless, these 3 approaches constitute the most frequently used craniotomies to access anterior skull base tumors, depending on tumor anatomy and location.

Although literature exists comparing the differences across the approaches in specific tumor locations,^6,7^ to the best of our knowledge, no study has comprehensively evaluated the role of craniotomy approaches on outcomes after resection of large anterior skull base meningiomas.^4^ As such, our study aims to compare the outcomes of the frontal, bifrontal, and pterional approaches in outcomes after the resection of large anterior skull base meningiomas.

## METHODS

### Study Design

This was a retrospective study at a single institution between January 2010 and June 2024. All patients were identified using a formal request on the electronic medical record system. This study was registered as a formal audit following institutional and departmental research and ethics committee approval (identifier: NSG_06). All included patients consented to their procedure and for anonymized, relevant data to be used within institutional retrospective studies.

### Participants

All patients were required to undergo primary resections of large anterior skull base meningiomas, defined as a meningioma situated within the anterior cranial fossa measuring ≥3 cm. Sellar and pituitary tumors were not included within this study. All patients were 18 years or older and were required to have ≥1-year of follow-up data after resection. All patients also underwent bifrontal, frontal, or pterional craniotomies. Patients who underwent other types of craniotomies (orbito-zygomatic and supraorbital) were excluded from the analysis.

### Outcomes

Following identification of all patients, 5 individuals collected longitudinal data from patient charts, surgical documents, and outpatient follow-up results. The following outcomes were extracted:

Baseline demographic factors (age, sex, deprivation, Karnofsky Performance Status [KPS]), tumor characteristics (size, location, grade, consistency, surrounding oedema), surgical characteristics (duration of surgery, length-of-stay, approach, extent of resection, complications, reoperations, adjuvant therapy), outcomes (KPS, cosmetic outcomes, discharge), survival outcomes (time to complications, progression-free survival [PFS], overall survival [OS]), and follow-up data.

Tumor consistency was classified using the system established by Zada et al^8^ (2013). Complications were classified using the Landriel-Ibañez classification,^9^ with minor complications encompassing grade 1 complications and major complications encompassing grade 2 complications and above (**Supplementary Table 1**, http://links.lww.com/ONS/B264). Cosmetic outcomes were identified through outpatient records, with an expressed discontent with the cosmetic outcomes (unamenable to repair) classified as “unfavorable.”

### Comparisons

After grouping into 3 cohorts based on approach, any significantly different baseline characteristics across 1 or more groups were used to perform propensity-score matching (PSM) using a multinomial logistic regression model with a one-to-many nearest neighbor algorithm. In our study, PSM was performed with respect to tumor size, grade, and preoperative KPS.

### Statistical Analysis

To compare both baseline characteristics and outcomes after resection, the Shapiro-Wilk test, paired *T*-test, and analysis of variance test were used. Tukey test analyses were used to depict descriptive statistics, with outliers depicted with their specific data points. Standardized mean differences (SMDs) were calculated before and after matching to assess balance, with an |SMD| <.1 considered indicative of adequate balance. Kaplan-Meier (KM) curves were generated to document complication and survival profiles across the 3 groups after resection, with the log-rank (Mantel-Cox) test used to identify significant differences in survival across groups. Multiple-linear regressions were performed using a series of other potential predictors to identify their role on complication, progression, and survival across the follow-up period, across all 3 approaches.

## RESULTS

**FIGURE 1.**
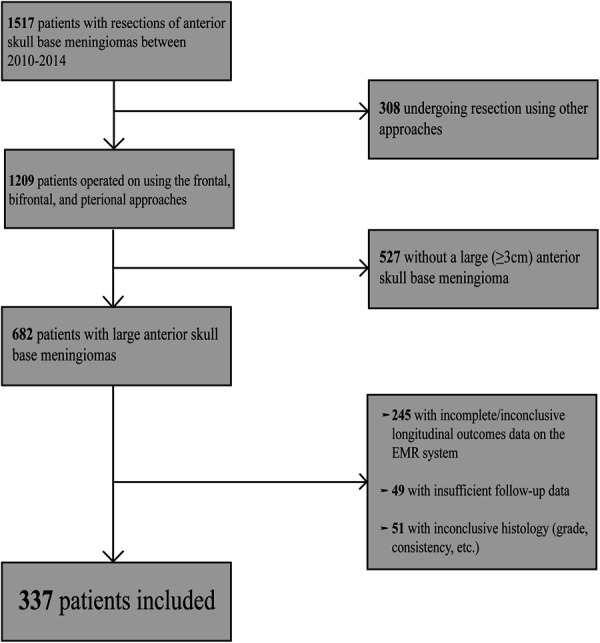
Flowchart of included and excluded patients. EMR, electronic medical record.

In total, 337 patients (140M, 197F) made up the final sample (Figure 1), with a mean age of 57.70 (14.34) years. Within this sample, 80 patients underwent bifrontal craniotomies, while 189 and 68 underwent frontal and pterional craniotomies, respectively. The mean follow-up of all patients was 36.02 (13.77) months after resection. Further demographic characteristics of participants are presented in Table 1.

**TABLE 1.** Baseline and Postoperative Demographic Characteristics of Included Participants

Variable	Value	Significantly different across the 3 approaches?
Sex		
M	140 (41.5%)	No (*P* = .8912)
F	197 (58.5%)	
Age (mean, SD, y)	57.70 (14.36)	No (*P* = .1418)
Deprivation decile (median, IQR)	5 (4-7)	No (*P* = .8172)
Tumor size (mean, SD, cm)	5.02 (1.38)	**Yes (*P* = .0021)**
Preoperative Karnofsky Performance Status (median, IQR)	80 (70-90)	**Yes (*P* = .0059)**
Tumor grade (median, IQR)	1 (1-2)	**Yes (*P* = .0025)**
Surrounding oedema	35 (10.4%)	No (*P* = .1171)
Laterality		
Left predominant	159 (47.2%)	No (*P* = .1812)
Right predominant	178 (52.8%)	
Location		
Olfactory Groove	122 (36.2%)	No (*P* = .0839)
Planum Sphenoidale	101 (30.0%)	No (*P* = .0891)
Sphenoidal Wing	57 (16.9%)	No (*P* = .1615)
Clinoidal	49 (14.5%)	No (*P* = .2615)
Other	8 (2.4%)	No (*P* = .9719)
Approach used		
Bifrontal	80 (23.7%)	—
Frontal	189 (56.1%)	—
Pterional	68 (20.2%)	—
Extent of resection		
Subtotal	62 (18.4%)	No (*P* = .1618)
Near-Total	143 (42.4%)	No (*P* = .6200)
Gross Total	132 (39.2%)	No (*P* = .1736)
Landriel-Ibañez classification of complications		
Grade 1	43 (12.7%)	**Yes (*P* = .0291)**
Grade 2	19 (5.6%)	No (*P* = .7219)
Grade 3	8 (2.4%)	No (*P* = .5757)
Grade 4	0 (0.0%)	No (*P* = .1231)
Adjuvant therapy		
No	332 (98.5%)	No (*P* = .7623)
Yes	5 (1.5%)	
Postdischarge destination		
Home	274 (81.3%)	No (*P* = .1818)
Rehabilitation Facility	12 (3.6%)	No (*P* = .5443)
Another ward/hospital	38 (11.3%)	**Yes (*P* = .0167)**
Hospice/end-of-life care	3 (0.9%)	No (*P* = .4219)
Unclear	10 (3.0%)	No (*P* = .8660)

Bold: reached statistical significance (*P* < .05).

**FIGURE 2.**
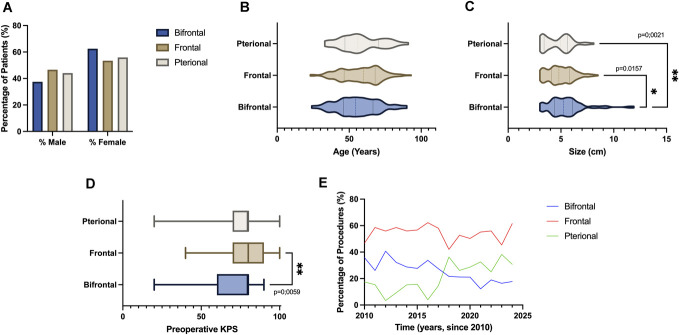
Baseline (pre–propensity-score matching) differences in gender **A**, age **B**, tumor size **C**, and preoperative KPS **D** across the bifrontal (n = 80), pterional (n = 68), and frontal groups (n = 189), alongside usage of approaches over time at our institution **E**. KPS, Karnofsky Performance Status.

No significant differences were observed in the distribution of overall gender (Figure 2A) or age (Figure 2B) across the 3 groups of patients. The patients undergoing bifrontal craniotomies were found to have a significantly larger mean tumor size (5.46 cm) vs the frontal (4.96 cm, *P* = .0157) and pterional (4.70 cm, *P* = .0021), respectively (Figure 2C). Moreover, patients in the frontal group were found to have a significantly higher mean tumor grade vs the pterional (1.65 vs 1.23, *P* = .0025) group, with no other significant differences found. The frontal group was found to have a significantly higher mean KPS (Figure 2D) vs the bifrontal group (77.17 vs 71.15, *P* = .0059). Finally, we found a higher usage of the pterional approach over time at our institution, contrasted by reduced usage of the bifrontal approach (Figure 2E). No significant differences were found in relation to tumor consistency, location, laterality, surrounding oedema, or adjuvant therapy (Table 1). Patients in the bifrontal group also experienced a greater risk of grade 1 complications (17.5%, *P* = .0291) and discharge to another ward/hospital (21.25%, *P* = .0167).

Based on preoperative differences in baseline characteristics, PSM was performed based on tumor size, grade, and preoperative KPS. In our sample, this resulted in a matched data set of 33, 96, and 29 patients in the bifrontal, frontal, and pterional groups, respectively, with a total sample of 158 matched patients. The PSM was highly successful in identifying a comparable data set of patients, with |SMD|<.1 achieved 8 of the 9 comparisons, compared with only 1 of 9 comparisons without PSM (Table 2).

**TABLE 2.** SMDs of All Non-PSM and PSM Comparisons

Variable	Comparison	SMD	|SMD| < 0.1
Without propensity-score matching			
Size	Bifrontal vs frontal	0.5025	No
	Bifrontal vs pterional	0.7619	No
	Frontal vs pterional	0.2594	No
Grade	Bifrontal vs frontal	−0.1156	No
	Bifrontal vs pterional	0.2619	No
	Frontal vs pterional	0.3775	No
Preoperative KPS	Bifrontal vs frontal	−0.3191	No
	Bifrontal vs pterional	−0.0951	Yes
	Frontal vs pterional	0.224	No
With propensity-score matching			
Size	Bifrontal vs frontal	0.0832	Yes
	Bifrontal vs pterional	0.0631	Yes
	Frontal vs pterional	−0.0200	Yes
Grade	Bifrontal vs frontal	0.0823	Yes
	Bifrontal vs pterional	−0.0405	Yes
	Frontal vs pterional	−0.1628	No
Preoperative KPS	Bifrontal vs frontal	0.0881	Yes
	Bifrontal vs pterional	0.0016	Yes
	Frontal vs pterional	−0.0726	Yes

KPS, Karnofsky Performance Status; PSM, propensity-score matching; SMD, Standardized mean difference.

**FIGURE 3.**
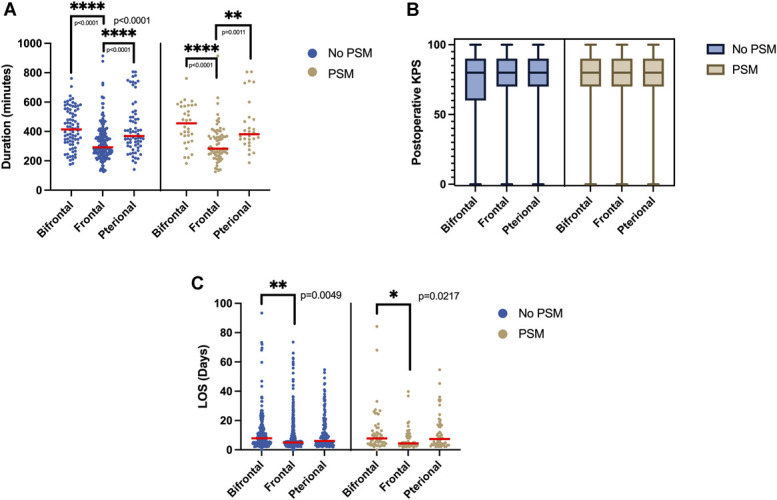
Differences in surgical duration **A**, KPS at discharge **B**, and length-of-stay **C** across the bifrontal (n = 80), pterional (n = 68), and frontal groups (n = 189), both before and after PSM. KPS, Karnofsky Performance Status; PSM, propensity-score matching.

Before PSM, patients undergoing frontal craniotomies were found to have a significantly lower mean duration in minutes (325.6) vs the bifrontal (422.6, *P* < .0001) and pterional (424.4, *P* < .0001) groups. After PSM, patients in the frontal groups continued to display significantly lower mean surgical durations (316.7) vs the bifrontal (448.4, *P* < .0001) and pterional (436.9, *P* = .0011) groups, respectively (Figure 3A). No significant differences were observed in postoperative KPS across groups (Figure 3B). We found a significantly higher mean length-of-stay (Figure 3C) in the bifrontal approach vs the frontal approach (12.28 vs 7.03 days, *P* = .0217).

**FIGURE 4.**
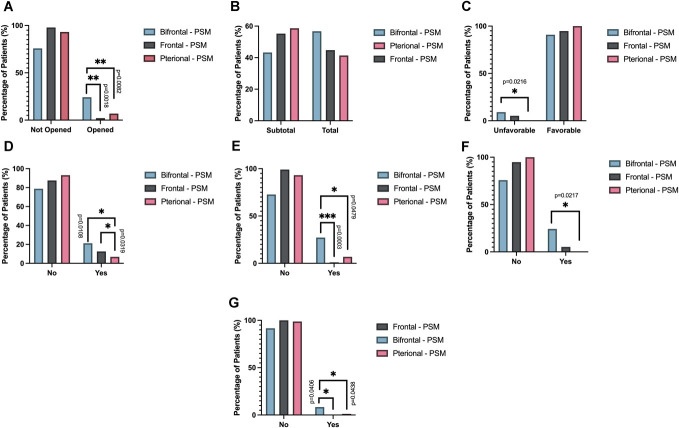
Differences in frontal sinus entry **A**, extent-of-resection **B**, cosmetic outcomes **C**, medical complications **D**, surgical complications **E**, reoperations **F**, and cerebrospinal fluid leaks **G** across the bifrontal (n = 36), pterional (n = 29), and frontal groups (n = 96), after PSM. PSM, propensity-score matching.

The bifrontal group was significantly more likely to have the frontal sinus breached intraoperatively (20.0%) vs the frontal (2.1%, *P* < .0001) and pterional (4.4%, *P* < .0001) groups, with these differences persisting after PSM (Figure 4A). No significant differences were found in the proportion of patients with gross total resection across the groups (Figure 4B). Patients in the bifrontal group were significantly more likely to report unfavorable cosmetic outcomes vs other groups (6.25%, *P* = .0054), both before PSM and after PSM (Figure 4C).

The bifrontal group reported a significantly higher proportion of medical complications vs the pterional (25.0% vs 7.35%, *P* < .0001) and frontal (25.0% vs 11.1%, *P* = .0028) groups, respectively, which was also significant after PSM (Figure 4D). The bifrontal group also reported a significantly higher proportion of surgical complications vs the pterional (22.5% vs 8.82%, *P* = .0031) and frontal (22.5% vs 4.2%, *P* < .0001) groups, respectively, with these differences also observed after PSM (Figure 4E). The bifrontal group also reported a significantly higher reoperation rate vs the frontal (17.5% vs 5.9%, *P* = .0109) and pterional (17.5% vs 4.4%, *P* = .0391) groups. After PSM, the bifrontal group continued to show significantly higher reoperation rates vs the pterional group (12.1% vs 0%, *P* = .0217) (Figure 4F). Finally, the bifrontal group was also significantly more likely to report cerebrospinal fluid (CSF) leaks (8.2%) vs the frontal (*P* = .0406) and pterional (*P* = .0438) groups after PSM (Figure 4G).

**FIGURE 5.**
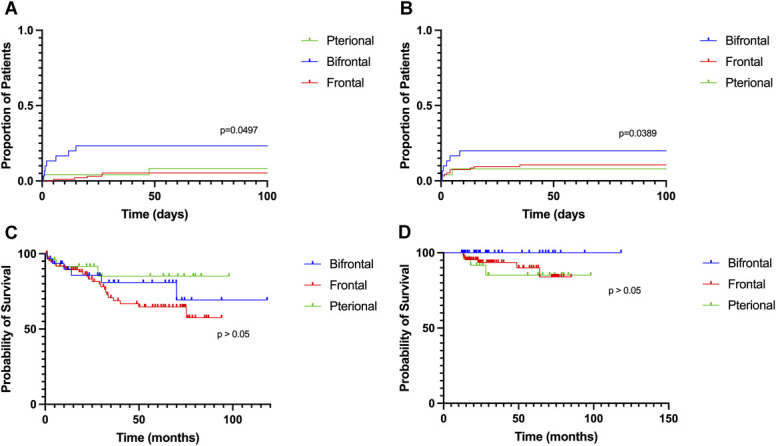
Time to minor complications **A**, time to major complications **B**, progression-free survival **C**, and overall survival **D** across the bifrontal (n = 36), pterional (n = 29), and frontal groups (n = 96), after propensity-score matching.

Within the KM analysis, after PSM, the bifrontal group was found to have a significantly lower time to minor complications vs the frontal group (*P* = .0497). Although the bifrontal group was also found to have a significantly shorter time to major complications vs the frontal (*P* = .0389) group, this was no longer observed following PSM. With respect to PFS, the frontal group was found to have a significantly lower median PFS (88.6 months) vs the pterional groups (*P* = .0048), with no significant differences after PSM. No significant differences were observed in OS across the 3 groups, both before and after PSM. These findings are presented in Figure 5.

Finally, to evaluate other factors unaccounted for in our analysis, we conducted multiple linear regressions to identify other baseline variables that were directly associated with complications, PFS, and OS, alongside comparing outcomes across the three groups to identify potential interactions between predictors and approaches (Table 3). For the pterional group, we found that olfactory groove tumors were associated with a significantly worse OS across the follow-up period (β0 = −3.143, 95% CI [−7.079, −0.4419]). For the frontal group, we found that lower extent-of-resection was associated with a greater risk of minor complications (β0 = −1.627, 95% CI [−0.1656, −3.363]). No other significant predictors were found for patients undergoing bifrontal craniotomies or across the groups.

**TABLE 3.** Multiple Logistic Regressions of Tumor Grade, Extent-of-Resection, Laterality, Surrounding Oedema, and Tumor Location on Outcomes Across Approaches

Approach	Predictor	Minor complications		Major complications		PFS		OS	
		β	95% CI	β	95% CI	β	95% CI	β	95% CI
Bifrontal	Intercept	1.423	−1.349 to 4.403	−2.546	−6.866 to 0.5012	0.8887	−1.646 to 3.718	2.266	−0.8210 to 6.665
	Grade	0.2261	−0.2103 to 0.6625	−0.04534	−0.4851 to 0.3944	0.1261	−0.3103 to 0.5625	−0.1584	−0.6021 to 0.2853
	Extent-of-resection	−1.658	−4.381 to 0.3742	0.7960	−1.203 to 2.981	−0.3710	−2.076 to 1.362	0.4728	−1.701 to 2.851
	Left-sided	1.647	−0.6621 to 4.575	1.243	−1.031 to 4.074	−0.9551	−2.957 to 0.9042	−2.291	−6.409 to 0.4117
	Oedema	−0.3640	−3.691 to 2.287	1.837	−0.8445 to 5.263	0.9864	−1.589 to 4.232	−2.074	−5.608 to 0.6506
	Olfactory Groove	−0.8731	−2.611 to 0.7403	−1.493	−3.407 to 0.2128	0.2108	−1.378 to 1.824	1.000	−0.7341 to 2.963
	Planum Sphenoidale	−1.155	−3.369 to 0.8021	1.752	−0.2435 to 4.161	−0.7364	−2.614 to 1.048	−1.093	−3.272 to 0.9141
	Sphenoidal Wing	−1.085	−3.158 to 0.7040	0.3047	−1.526 to 2.228	−0.09614	−1.769 to 1.521	−0.9874	−3.124 to 0.9006
	Clinoidal	−1.466	−3.728 to 0.4554	1.301	−0.7586 to 3.883	−0.3105	−2.184 to 1.436	0.7935	−1.248 to 2.923
Pterional	Intercept	1.532	−1.677 to 5.484	1.586	−2.644 to 6.370	−0.5472	−3.680 to 2.520	0.1132	−4.398 to 5.044
	Grade	−0.7101	−1.321 to 0.09873	0.2202	−0.3929 to 0.8333	0.1218	−0.4882 to 0.7319	0.1603	−0.4291 to 0.7498
	Extent-of-resection	1.228	−0.5489 to 3.277	1.655	−0.5794 to 4.866	−0.3054	−2.225 to 1.445	−1.730	−4.442 to 0.4289
	Left-sided	−1.013	−3.454 to 1.133	−1.638	−5.327 to 0.9134	0.9223	−1.122 to 3.334	0.07348	−2.768 to 3.103
	Oedema	−0.6794	−2.825 to 1.289	2.712	−0.6806 to 7.009	−0.8600	−2.932 to 1.120	0.6203	−2.145 to 3.498
	Olfactory Groove	−0.9237	−3.351 to 1.370	−2.442	−6.631 to 0.5405	0.1864	−2.129 to 2.566	−**3.143**	−**7.079 to** −**0.4419**
	Planum Sphenoidale	0.2619	−1.895 to 2.352	1.150	−1.656 to 4.575	−1.416	−3.764 to 0.5860	3.194	0.3771 to 7.510
	Sphenoidal Wing	−0.3809	−2.986 to 2.117	−2.240	−6.689 to 0.7698	1.652	−0.8684 to 4.677	−2.415	−6.640 to 0.6669
	Clinoidal	0.1344	−1.692 to 1.969	−2.301	−6.090 to 0.1572	0.6361	−1.135 to 2.546	2.843	0.3561 to 6.762
Frontal	Intercept	−**2.376**	−**4.920 to** −**0.09843**	−2.416	−5.473 to 0.2416	−1.356	−3.118 to 0.3084	−2.089	−4.890 to 0.4434
	Grade	−0.09411	−0.5783 to 0.3900	−0.05800	−0.5753 to 0.4593	0.2048	−0.1654 to 0.5750	0.6708	−0.1944 to 1.347
	Extent-of-resection	−**1.627**	−**0.1656 to** −**3.363**	−0.8224	−2.583 to 0.7283	−0.6747	−1.746 to 0.3401	−1.101	−3.140 to 0.5504
	Left-sided	−0.8329	−2.201 to 0.4654	0.3377	−1.049 to 1.846	0.2563	−0.6922 to 1.244	0.3498	−1.160 to 2.028
	Oedema	−0.8300	−2.839 to 0.7346	0.4706	−1.117 to 1.992	0.1723	−0.9121 to 1.220	−0.4283	−2.472 to 1.227
	Olfactory Groove	0.4566	−1.028 to 2.031	−1.166	−2.857 to 0.2790	0.2210	−0.8153 to 1.277	0.1607	−1.525 to 1.923
	Planum Sphenoidale	−0.1537	−1.627 to 1.294	0.5447	−1.230 to 2.625	−0.04047	−1.118 to 1.038	−0.02573	−1.751 to 1.772
	Sphenoidal Wing	0.3656	−1.010 to 1.761	0.9279	−0.5086 to 2.504	0.1718	−0.8378 to 1.179	−0.3080	−2.040 to 1.277
	Clinoidal	−0.4847	−1.920 to 0.9003	−0.03078	−1.505 to 1.428	0.4672	−0.5033 to 1.473	0.1266	−1.442 to 1.704

OS, overall survival; PFS, progression-free survival.

Bold: reached statistical significance (*P* < .05).

## DISCUSSION

This study presents novel findings regarding the role of the frontal, bifrontal, and pterional craniotomies on patient outcomes following large anterior skull base meningioma resection. Size, grade, and preoperative KPS were significantly different across groups, suggesting that choice of approach was likely dependent on these characteristics. Larger tumors were more frequently resected using the bifrontal approach, likely due to a wider operative field, supported by the literature which highlights the role of tumor size and location in determining surgical strategy.^6^ Owing to the link between symptom burden and increased tumor size, larger tumors at baseline in the bifrontal group may have contributed to reduced preoperative KPS.^10^ The frontal group had a significantly higher tumor grade compared with pterional group, potentially due to more aggressive tumors allowing detection at a relatively lower size compared with lower grade tumours.^11,12^

We found that the bifrontal and pterional approaches had significantly longer surgical durations than the frontal approach—likely due to differences in access/surgical complexity. Bifrontal craniotomies offer direct tumor access but require larger incisions and bone removal, contributing to longer operations.^13^ Owing to larger tumors being resected more frequently using the bifrontal approach, more time may be needed to optimize resection and minimize injury.^14^ Similarly, the pterional approach also requires care in avoiding major neurovascular structures.^4,15^ Our findings hence suggest that the direct access offered by frontal craniotomies may call for a faster operation.

Patients in the bifrontal group were significantly more likely to have their frontal sinus breached intraoperatively, alongside reporting worse cosmetic outcomes vs other groups. The bifrontal approach, through its wider incision and greater tissue manipulation, may increase the risk of infection, oedema, and brain injury from retraction.^14,16,17^ Second, entry into the frontal sinus is associated with a greater risk of CSF leakage,^15^ which was more frequent in bifrontal craniotomies. By contrast, the less invasive nature of the pterional and frontal approaches may lead to fewer complications and better cosmetic outcomes.^4^

After PSM, medical and surgical complications remained significantly higher in the bifrontal group. Minor complications included non–life-threatening deviations from expected postoperative course, while major complications included interventional treatment, intensive care unit admission, or death (**Supplementary Table 1**, http://links.lww.com/ONS/B264).^9^ The higher complication risk in the bifrontal approach may result from multiple factors, including longer surgical duration,^18^ a greater risk of CSF leaks,^15^ and the higher risk of infection due to the wider incision and greater tissue manipulation.^14^

Within the KM analysis, the bifrontal group reported significantly shorter times to and higher proportions of minor/major complications vs the other groups, possibly attributed to the wider ear-to-ear access.^19,20^ PFS was significantly worse in the frontal group, particularly before PSM. Since we found that the frontal group reported a higher baseline grade, they may have been predisposed to progression. Moreover, studies highlight the usage of frontal approach for symptomatic control (through debulking) as opposed to for curative intent vs other approaches in other tumor types,^21^ potentially resulting in increased recurrence and lower PFS. Finally, OS was comparable across the 3 groups. This implies that despite higher rates of complications, the OS for patients having undergone bifrontal craniotomy is similar to other approaches,^14^ as survival is often most dependent on tumor histology, which may not play as significant of a prognostic role in OS in a meningioma cohort.^14,15^

Additional multivariable modelling highlighted that approach-specific factors may influence outcomes beyond the variables addressed through matching. In the pterional cohort, olfactory groove tumors were independently associated with a higher risk of mortality, potentially resulting from their deep midline location and proximity to the anterior cerebral arteries and optic apparatus, increasing surgical complexity and postoperative neurological compromise.^10^ In the frontal cohort, subtotal resection was significantly associated with more frequent minor complications, underscoring the importance of achieving gross total resection when safe to do so. No other independent predictors were identified (including grade), suggesting that while |SMD|≤.1 was not achieved for one comparison of grade during PSM, this disparity did not seem to have a direct effect on biasing the group-specific outcomes and PFS/OS outcomes. We also did not find any significant difference in predictors such as laterality or surrounding oedema on patient complications, PFS, and OS, suggesting that the differences we observed in patient outcomes and the KM analysis can be more directly attributed to the approach itself, as opposed to differences in preoperative patient-factors.

Although our results showed detriments of the bifrontal and frontal approaches for complication rates and PFS, respectively, these findings should not be interpreted as evidence for reduced usage of these approaches. Rather, the optimal approach is situational and depends on tumor and patient-specific factors. In our institution, the bifrontal approach was preferentially used for large, often bilateral/midline lesions, where maximal exposure is essential and risk of neurovascular injury with lateral approaches is heightened. The frontal approach was more frequently selected for smaller, high-grade tumors where rapid access and aggressive resection were feasible, which may be appropriate in younger patients if durable PFS is achievable through total resection. The pterional approach was favored for lesions with significant lateral extension, best approached through the Sylvian fissure. Notably, pterional craniotomies for olfactory groove meningiomas were associated with worse OS, underscoring that lateral approaches may not always be advantageous for deep midline tumors. Thus, in practice, approach selection balances surgical exposure and preservation of function against the anatomic complexity and anticipated risk.

### Limitations

This study was limited by its retrospective design and introduces the potential for selection bias. We also identified variables such as KPS and tumor consistency based on retrospective data, which may not fully reflect patients' actual preoperative and postoperative status. In addition, changes in surgical techniques and methods over time were not accounted for, which may have influenced outcomes. Since our cohort also included resections performed by multiple surgeons at our institution, surgeon expertise may be a confounding factor, which may affect outcomes such as extent-of-resection and complications. Another significant challenge concerning the results of this study is whether tumors typically treated with one approach can be safely resected using another. Even if one method seems superior in outcomes, anatomic constraints and surgeon expertise may limit the ability to switch from a traditionally used approach to an alternative. Notably, this study did not include patients treated with endoscopic approaches. Research has suggested increasing usage of this approach in the management of anterior skull base tumors, alongside substantial technological and surgical improvements in execution.^22^ As such, future work that comparing open surgical approaches vs endoscopic approaches to anterior skull base meningiomas may be beneficial. Our regression analysis also found significant roles of further factors (that were nonsignificant at baseline) such as tumor location and extent-of-resection as predictors of outcomes, beyond those adjusted for in the PSM algorithm. This suggests that although the matched groups were highly comparable, other subtle differences may have remained across the groups, limiting the generalizability of our findings. Although these factors could not be included in the PSM algorithm, owing to their lack-of-significance at baseline and significant downstream reductions in postmatching sample size, further work with larger samples may benefit from their inclusion. Finally, although PSM was largely effective, we were unable to achieve a comparable level of similarity concerning tumor grade. This could introduce some level of baseline bias resulting from differences in baseline grade across groups, affecting the precision of the matched comparisons and highlighting the complexity of tumor biology beyond simple grading systems.

A variety of patients also had missing information on records and histology reports, leading to the exclusion of potentially useful but fragmented data. This could result in potential selection bias, as patients with more complete records may have been more likely to be included, which could be reduced with future multicenter work with broader inclusion criteria. Moreover, we had significant variations in follow-up duration across patients. Although the mean follow-up was around 3 years, some patients had significantly shorter follow-up periods. This may have also limited the ability to assess long-term outcomes such as PFS and OS consistently, especially with certain patient groups achieving subtotal resection, and hence being more prone to recurrence longitudinally. As such, it is likely that our reported PFS and OS outcomes may differ over longer follow-up windows. This study's retrospective nature also prevents causality from being established. Although significant associations between surgical approaches and outcomes were observed, these findings do not imply that one approach directly leads to better or worse results, with other unmeasured predictors potentially also playing significant roles. Finally, PSM resulted in a reduction in sample size, decreasing the study's statistical power.

## CONCLUSION

Our study found that the bifrontal group reported significantly more frequent complications alongside worse cosmetic outcomes. KM analysis revealed a significantly higher incidence of minor complications in the bifrontal group, alongside reduced PFS in the frontal group. Finally, regression analyses revealed subtotal resection and tumor location played substantial roles in approach-specific outcomes, providing key insights about the role of operative technique and approach selection. Through these findings, we hope this study can provide clinicians with an increased understanding of the role of approach choice on outcomes across patients.

## Supplementary Material

## SUPPLEMENTAL DIGITAL CONTENT

**Supplementary Table 1**. Landriel-Ibañez Classification of Complications.

## COMMENTS

The authors conducted a retrospective study involving 337 patients with large anterior skull base meningioma. 80, 189, and 68 patients of them received surgery through bifrontal (BF), frontal (F) and pterional (P) craniotomies, respectively. Propensity-score matching underwent to achieve 3 groups of similar patients, based on size, grade, and preoperative KPS. The BF group showed significantly higher frequency of medical and surgical complications, alongside significantly worse cosmetic outcomes, compared to other groups. KM analysis showed significantly higher rate of minor complications in the BF group, alongside reduced progression-free survival (PFS) in the F group. I have some queries regarding as below. Although P was associated with fewer complications than BF and higher PFS than F, the authors did not mention P is universally “best.” The outcome of P was not superior in all contexts. For example, the regression analysis showed resection of olfactory groove meningiomas via P was associated with worse OS, highlighting that lateral approaches may be less favorable for deep midline tumors. Each approach has specific advantages and disadvantages depending on the tumor characteristics. The difference in PFS of F was only observed prior to matching. We should confirm that the approach selection should be tailored to tumor size, location, extension, grade, and patient-specific considerations. In addition, as the authors addressed in the text, the number of BF has been decreasing because of the rapid development of endoscopic trans-nasal or endoscopic supraorbital keyhole approach. Further investigation will be required to compare open surgery and endoscopic approaches.

**Satoshi Kawajiri and Kenichiro Kikuta**

*Fukui, Japan*
