# Supplementary material for: Comparative Outcomes of Frontal, Bifrontal, and Pterional Craniotomies for Resection of Large Anterior Skull Base Meningiomas
Source: Oper Neurosurg. 2025 Nov 28;31(3):484–94. doi: 10.1227/ons.0000000000001830 (PMC13456562; doi:10.1227/ons.0000000000001830)
Supplement: Supplementary file 1 [file ons-31-484-s001.docx]

**Supplementary Table 1:** Landriel-Ibañez Classification of Complications

| Grade | Subtype | Description |
| --- | --- | --- |
| **Grade 1** (any non-life-threatening deviation from normal postoperative course, not requiring invasive treatment) | 1A | Complication requiring no drug treatment |
|  | 1B | Complication requiring drug treatment |
| **Grade 2** (complication requiring invasive treatment) | 2A | Complication requiring intervention without general anaesthesia |
|  | 2B | Complication requiring intervention with general anaesthesia |
| **Grade 3** (Life-threatening complications requiring management in ICU) | 3A | Complication involving single organ failure |
|  | 3B | Complication involving multi-organ failure |
| **Grade 4** | | Complication resulting in death |
